# Neurorehabilitation: Looking Back and Moving Forward, 1st Edition

**DOI:** 10.3390/healthcare11101452

**Published:** 2023-05-17

**Authors:** Grigorios Nasios, Lambros Messinis, Efthimios Dardiotis, Markos Sgantzos

**Affiliations:** 1 Department of Speech & Language Therapy, School of Health Sciences, University of Ioannina, 45500 Ioannina, Greece; 2 Laboratory of Cognitive Neuroscience, School of Psychology, Aristotle University of Thessaloniki, 54124 Thessaloniki, Greece; lmessinis@psy.auth.gr; 3 Department of Neurology, Faculty of Medicine, University of Thessaly, 41110 Larissa, Greece; ebsdar@gmail.com; 4 Department of Anatomy, Faculty of Medicine, University of Thessaly, 41110 Larissa, Greece; sgantzosmarkos@gmail.com

Rehabilitation is “a set of interventions designed to optimize functioning and reduce disability in individuals with health conditions in interaction with their environment”, according to the recent report from the World Health Organization (WHO), released in January 2023 [1]. As was pointed out in this report, rehabilitation is not “a luxury health service”, but rather an “investment, with cost benefits for both the individuals and society”, and potentially all of us need it, or will need it. As it is estimated, globally, about 2.4 billion people are currently living with a health condition that may benefit from rehabilitation [1], many of them with neurological diseases, to whom neurorehabilitation services should be offered. As was stated in our proposal, when releasing this Special Issue, progress in neurorehabilitation has been impressive over the years, but mainly in the last two decades. It follows the progress of clinical neurosciences in general, which is accelerated by several factors, technology being a major factor. Moving forward from empirical approaches, to an era of evidence-based practice, in which multidisciplinary approaches and translational research are applied even from the acute phase, through the disease course, numerous institutes and research groups have attempted to illuminate how nervous system plasticity and reorganization dynamics can be manipulated to offer better treatments, mainly non-pharmacological and neurobehavioral treatments. The lack of pharmaceutical remedies for the management of such disorders and the importance of cognitive or neuropsychological rehabilitation, neuromodulation, and multidisciplinary approaches were the focus of our attention in another recent Special Issue [2]. This fast-changing landscape of neurological rehabilitation, posing the need for a close follow up of the literature, and updated guidelines, was underlined via numerous examples and three of them are briefly reported below.

In 2019, we published a review article entitled “From Broca and Wernicke to the Neuromodulation Era…”, focusing on “methods for coupling new knowledge regarding the functional reorganization of the brain with sophisticated techniques capable of activating the available supportive networks in order to provide improved neurorehabilitation strategies” for recovery from aphasia [3]. Since then, low frequency repetitive transcranial stimulation (LF-rTMS) of the right inferior frontal gyrus (IFG) holds level B evidence for use in chronic poststroke non-fluent aphasia [4]. Furthermore, many investigators worldwide have published and currently are running protocols applying non-invasive transcranial magnetic or electrical stimulation, under different scenarios, regarding the brain region(s) targeted, the nature of lesions (vascular or neurodegenerative), and the time after aphasia onset.

A second example is rehabilitation of cognitive impairment in people living with multiple sclerosis (pwMS). While “no evidence” to support cognitive rehabilitation was the conclusion of a review of the literature in 2012 [5], there was a turn to “some evidence” in 2016 [6], with the “U-turn” completed in 2021, when the same authors changed their recommendation to “there is evidence” to support the rehabilitation of memory in pwMS [7]. It must be noted here that within “only” ten years of research progress, clinical practice was revolutionized.

A third example is neglect; even though it is a frequent consequence of right hemispheric damage [8], with a heavy impact on prognosis, and despite the existing evidence supporting its rehabilitation [4], it seems that many of us “neglect” it, being highly underdiagnosed and untreated [9]. In this case, it appears that the research has failed to “inform” clinical practice, at least not widely.

In this Special Issue of *Healthcare*, we present eleven novel articles from international groups of colleagues, including five clinical studies, five review articles, and one brief report.

Mariacristina Siotto et al. studied gender differences in 61 subacute stroke patients in terms of oxidative stress status, and the possible correlation between biomarkers of oxidative stress status and motor impairment, disability, and pain [10]. Measurements of hydroperoxide levels (d-ROMS), antioxidant activity (BAP test) and relative antioxidant capacity (OSI index) glucose levels and the lipid profile of the patients were performed. High levels of d-ROMS were recorded in all the patients, independently of gender, while BAP levels were in the normal range. Statistically significant gender differences were noted in the BAP and OSI values, with women having lower values, while in the male group, there was a negative correlation between oxidative stress status and motor impairment. On the other hand, a completely different trend of correlation was observed in the female group, maybe due to unbalanced systemic oxidative stress. These gender differences in terms of oxidative stress status should trigger further research in post-stroke patients in rehabilitation settings.

The second article, hosted in this Special Issue, is an elegant research study by Wohno Choi, investigating the effectiveness of cognitive exercise therapy on upper extremity sensorimotor function and daily functioning in 30 patients with chronic stroke, randomly divided into an experimental group (*n* = 15) and control group (*n* = 15) [11]. The experimental group received a combination of cognitive exercise therapy and conventional occupational therapy, while the control group received conventional occupational therapy exclusively. The authors found that only the experimental group improved significantly in sensory function, motor function and activities of daily living, in contrast to the control group, which improved only in the domain of daily activity. They concluded that the combined application of cognitive exercise therapy and conventional occupational therapy seems to have stronger results in terms of improving sensory and motor functions in patients with chronic stroke.

Daniel Fernandez-Sanchis and his colleagues from Spain studied the cost-effectiveness of a single dry needling (DN) session in 23 patients with stroke in the chronic phase, divided into the intervention group (IG) and the sham group (SG) [12]. The two variables used for the cost-effectiveness study were the values of the EuroQol-5D questionnaire and the Modified Asworth Scale (MMAS) to obtain the percentage of treatment responders and the quality-adjusted life years (QALYs) for each alternative. The evaluation of costs was carried out from the prospective of the hospital, clinic, or health center. A favorable cost-effectiveness ratio of both the EUR/QALYs and EUR/responder for IG emerged from the data analysis. However, the sensitivity analysis did not confirm the dominance of dry needling (higher effectiveness with less cost) over sham dry needling. Through this study, the application of DN to the upper limb was highlighted as an affordable alternative method in patients with chronic stroke.

In a randomized controlled trial, Choi et al. investigated the clinical effectiveness of Backward or Forward Walking Training programs combined with motor tasks on the balance and gait function of children diagnosed with spastic hemiplegic cerebral palsy [13]. Twelve children were randomly assigned to the Forward Walking Training Group (FWT) (*n* = 6) and the Backward Walking Training Group (BWT) (*n* = 6). The exercise programs were conducted 3 times per week for 4 weeks with a duration of 40 min per session. After six weeks from the completion of the first intervention, crossover training was also conducted. There was a statistically significant improvement in the spatiotemporal gait parameters (velocity, step, and stride) in both groups. Additionally, statistically significant improvement was found in walking speed, and balance function; however, the improvement was more significant in the BWT group. The authors concluded the importance of including the BWT program to improve balance, and thus prevent fall injuries in this population.

Christos Bakirtzis and his colleagues from Greece performed a validation study evaluating the psychometric properties of the Greek version of the Multiple Sclerosis Work Difficulties Questionnaire-23 (MSWDQ-23) [14]. The study involved 196 patients with multiple sclerosis (MS), all full-time or part-time employees. In addition to completing the MSWDQ-23, participants also completed self-reported questionnaires on fatigue, mood, daily functioning, and quality of life. The study verified the three-factor structure of the Greek version of the MSWDQ-23. In addition, the convergent validity of the questionnaire was good, as greater difficulties in the work environment were associated with higher EDSS, lower performance in cognitive tests, more fatigue, anxiety, stress and depression, and worse quality of life. The internal consistency of the questionnaire was excellent. Through this study, the Greek version of the MSWDQ-23 was proven to be a valid tool, which can be used in the context of interventions aimed at improving the occupational status of people with multiple sclerosis.

In a review article, Polychronis et al. critically approached the relevant literature of drooling in patients with Parkinson’s disease (PD), and the possible relationship between excessive drooling and other clinical manifestations of PD, such as cognitive impairment, sleep difficulties, autonomic dysfunction, constipation, and orthostatic hypotension [15]. The study concluded that excessive drooling in PD patients is due to a wide range of factors. In parallel, excessive drooling was linked with a decrease in DAT binding in the striatum following DaTSCAN imaging.

Another review by Alexandratou et al. investigated the long-term neuropsychological effects following temporal lobe epilepsy surgery (a mean/median > 5 years post-surgery follow-up), according to the results of eleven included studies, based on the inclusion criteria defined in [16]. Interestingly, although one of the immediate consequences of surgery was a decline in cognitive functions, at the long-term follow-up, most studies demonstrated cognitive stability. Successful control of seizures leads mainly to the recovery of cognitive functions rather than a continued decline. The potential role of more selective surgery procedures in limiting cognitive side effects after surgery was also highlighted in this review.

Aloizou et al. reviewed the available studies that have used repetitive transcranial magnetic stimulation (rTMS) for the treatment of the most common types of dementia [17]. It has been argued that the application of this method, either alone or in combination with pharmaceutical therapy and cognitive training, appears to produce positive results in terms of improving cognitive functions. Although most protocols mostly involve the dorsolateral prefrontal cortex (DLPFC), it is a matter of further investigation. At the same time, the application of rTMS seems to benefit patients with mild cognitive impairment (MCI). Through this review, it can be perceived that rTMS is a promising method to improve the clinical picture of dementia, especially in Alzheimer’s disease. At the same time, this review will be a trigger for further research, which will investigate the potential effectiveness of rTMS not only in Alzheimer’s disease, but also in other types of dementia (vascular dementia—VD, Lewy body dementia—LBD; frontotemporal dementia—FTD).

Paulina Magdalena Ostroska and her colleagues from Poland reviewed the telerehabilitation of post-stroke patients in the era of the COVID-19 pandemic [18]. Their review evaluated the effectiveness of telerehabilitation on the functional status of post-stroke patients according to the results of 10 studies published from 2019 to 2021. Based on the studies that have investigated the feasibility and effectiveness of telerehabilitation programs in post-stroke patients, it has been argued that teletherapy appears to be effective not only in terms of functional recovery, but also in terms of mental and cognitive status, thereby improving the quality of life of patients. Through this review, the positive effects of telerehabilitation have been highlighted, which appear to be comparable to those of hospital treatment. Teletherapy appears to be an alternative method to traditional treatment and its applicability is particularly important in times of constraints, such as those caused by the SARS-CoV-2 pandemic.

In an observational study, Poveda-Garcia et al. investigated the ability to visualize mental motor images and the relationship between mental imagery and motor deficits of the upper limbs after stroke [19]. The study involved 39 patients, in whom upper limb movement and function, cognitive functions, and the ability to visualize mental images and functionality in daily life were assessed. The ability to visualize mental motor images of the upper limbs was highly significantly correlated with movement, functionality, and strength. In addition, visuospatial skills showed a correlation with the ability to visualize mental images, as well as with upper limb movement. Through this study, the particularly important role of the development of mental motor imagery in rehabilitation contexts is highlighted.

Finally, Agost-Gozalez et al. conducted a systematic review comparing the efficacy of percutaneous electrical stimulation (PTNS) and transcutaneous electrical stimulation (TTNS) procedures on the posterior tibial nerve in adults with idiopathic overactive bladder syndrome (OAB) who present with urinary incontinence (UI) [20]. A total of 19 studies published from 2015 to 2020 were included based on the inclusion criteria defined. The combined application of TTNS or PTNS procedures with other methods, such as pharmaceutical therapy or exercise, appears to be highly effective in reducing urinary incontinence (UI), and seems to contribute to a significant improvement in patients’ perception of quality of life. Regarding the comparison of the two treatments, PTNS and TTNS, both procedures seem to be equally effective, but TTNS is mainly preferred, as it is more comfortable for the patient. Through this review, not only two globally recognized treatment methods were compared but also possible combinations of these treatments with other methods were highlighted, which may bring about the best results in terms of treating adult OAB individuals with UI.

As is apparent to the reader, the articles published in this Special Issue cover a wide range of neurorehabilitation modalities, although they only represent a small proportion of the whole scene. We trust that they will assist clinicians to perform evidence-based interventions and illuminate the necessity of multidisciplinary collaboration. Furthermore, they share an important motivational drive, that is, the inspiration to move forward to a new era, where medical, basic research, engineering and high technology advances can develop more efficacious approaches, combining neuromodulation techniques under sophisticated neuroimaging guidance. These can be widely and remotely applied to all people that need them, organized under a fresh “patient-centric” approach, with home-based and tele-rehabilitation protocols replacing long stays in medical centers.

Of course, there are many issues that still need to be addressed and fulfilled. A major issue is the priority that nations in the world must give to rehabilitation; in the 2030 initiative, released in 2017, the WHO emphasized the largely unmet need for rehabilitation, since most people still have no access to such services, although they need them [21]. The initiative highlights the importance of strengthening health systems to provide rehabilitation, marking a new strategic approach for the global rehabilitation community, with ageing populations, and an increase in the number of people living with chronic disease [21].

Another major issue is the convincing results of both basic and clinical research; as we can clarify the underlying mechanisms of brain dysfunction during disease, we will also be able to understand the mechanisms under which interventions such as cognitive training, exercise, music, or neuromodulation can change the brain [22]. This could be a key factor in conducting clinical trials that test innovative rehabilitation treatments and result in convincing evidence to translate research into clinical practice, since the lack of empirical evidence remains one of the main limitations of this translation [23].

Closing this editorial, we would like to inform the reader of the new series of articles already published in the second edition of this Special Issue, and hope to provide our readers with further empirical evidence and points for discussion, once this new Special Issue is complete.
